# Repeat Intrathecal Triamcinolone Acetonide Application Reduces Acute Occurring Painful Dysesthesia in Patients with Relapsing Remitting Multiple Sclerosis

**DOI:** 10.1100/tsw.2006.86

**Published:** 2006-04-06

**Authors:** Kerstin Hellwig, Carsten Lukas, Niels Brune, Volker Hoffmann, Sebastian Schimrigk, Horst Przuntek, Thomas Müller

**Affiliations:** Department of Neurology, St. Josef Hospital, Ruhr University Bochum, Gudrunstrasse 56, 44791 Bochum, Germany

**Keywords:** relapsing remitting multiple sclerosis, triamcinolone acetonide, intrathecal, dysesthesia, therapy

## Abstract

We describe four patients with relapsing remitting multiple sclerosis (RRMS) who experienced a relapse with acute onset of painful sensations. Pain sensations disappeared in two of them and markedly reduced in the other ones after repeat application of intrathecal triamcinolone acetonide (TCA) following a prior unsuccessful treatment with intravenous steroids. TCA administration was well tolerated and no serious side effects occurred. Repeated intrathecal TCA injection may provide a substantial benefit in RRMS patients with acute onset of pain due to an inflammatory lesion within the spinal cord.

## INTRODUCTION

Various kinds of pain syndromes occur in patients with multiple sclerosis (MS) in the course of the disease[1,2,3]. Disability and spastic or neuronal damage may cause a wide variety of pain symptoms. However, the acute onset of pain symptoms may sometimes reflect the exacerbation of a MS relapse. Then the patients often experience, the Lhermitte's sign or painful neuralgia, which often results from an inflammatory process of the spinal cord in MS patients. The patients also characterize their pain sensations as circular in thoracic regions or in the limbs in addition to dysesthesia or as an increased painful sensitivity or even hyperpathy. Oral or intravenous steroid administration often provides no symptomatic benefit[4]. However, novel promising results suggest repeated intrathecal application of triamcinolone acetonide (TCA), a retarded-release steroid compound, as an additional therapeutic alternative in MS. Progressive MS patients with predominant spinal symptoms had a substantial short- and long-term therapeutic benefit according to open-label trials without relevant increase of cell injury markers in cerebrospinal fluid (CSF)[5,6,7]. These studies showed that six repeat intrathecal TCA injections within 3 weeks significantly reduced the expanded disability status scale (EDSS) score and accordingly increased the walking distance up to threefold. This achieved benefit remained stable with further single

TCA applications on a regular basis in an individually differing frequency every 6 to 12 weeks. This TCA treatment regimen was well tolerated and safe in all trials. Serious side effects, i.e., adhesive arachnoiditis or sterile meningitis, which temporarily caused a stop of research on the efficacy of intraspinal steroid application in MS, were not found in these studies[8]. The successful use of intrathecal TCA administration in MS patients who recently experienced an acute onset of pain syndromes prompted these case reports.

## SUBJECTS

Four relapsing remitting MS (RRMS) patients experienced a painful relapse that lasted at least 24 h and was not secondary to general infection[9]. All did not respond to a previous oral or intravenous steroid application before start of intrathecal TCA administration with the patients' written consent. No patient had a history of seizures, subdural hematoma, or severe postlumbar puncture syndrome. An atraumatic (Sprotte®) needle was employed for lumbar puncture and subsequent intrathecal TCA administration, in order to try to avoid onset of postlumbar puncture syndrome. Cell count and protein analysis in CSF was performed for safety reasons; 40 mg TCA were dissolved in 9 ml of isotonic saline solution under sterile conditions and then slowly injected over an interval of approximately 5 min. The patients were asked to stay in the supine position in bed for at least 6 h. This should reduce incidence of lumbar puncture syndrome and hypothetically support the diffusion of TCA in the CSF and the spinal cord[10,11]. A pre-existing, immune-system modulating drug therapy remained stable. The treatment effect on pain was evaluated with the use of the 11 point box scale (PBS), similar to a visual analog scale. This patient self-rating scale covers a range from 0–10 with 0 reflecting no pain and 10 maximum pain intensity. Patients were asked to assess their pain perception before the start and at the end of TCA administration. Clinical MS symptoms were additionally scored with the EDSS[12].

## Case 1

The 38-year-old female RRMS patient was hospitalized due to acute onset of painful dysesthesia 8 days before admission at the level of Th10. She had no immune modulating treatment. At admission, she also showed sensory transverse lesion of the spinal cord below L1, mild paraplegia, and ataxia (EDSS 3.5). Initially, we treated her with 1000 mg Solu Decortin® intravenously over 5 days. Symptoms of ataxia and paraplegia improved, however, the painful thoracic dysesthesia did not respond. Then TCA was administered on day 1 and day 4 after intravenous steroid treatment interval. The pain severity was 5 on the PBS before the start of the intrathecal TCA treatment and dropped to zero 1 day after the second TCA application. The EDSS score was 2.5 at that time. No side effects occurred; CSF cells and protein remained in normal range (see Table 1).

**TABLE 1**
**Findings in CSF During the TCA Treatment**

| N | Case 1 | Case 2 | Case 3 | Case 4 |
|---|---|---|---|---|
| 1 | 2/µl, 143 mg/l | 1/µl, 525 mg/l | 5/µl, 297 mg/l | 1/µl, 336 mg/l |
| 2 | 3/µl, 199 mg/l | 1/µl, 635 mg/l | 2/µl, 214 mg/l | 6/µl, 433 mg/l |
| 3 | | 1/µl, 473 mg/l | 1/µl, 179 mg/l | 1/µl, 226 mg/l |
| 4 | | 1/µl, 582 mg/l | | 1/µl, 192 mg/l |
| 5 | | 1/µl, 465 mg/l | | |

N = 1st, 2nd, 3rd, etc. TCA application, number of cells are given per µl (/µl), protein content in CSF is given in mg/l.

## Case 2

A 38-year-old female patient, suffering from RRMS since she was 6 years old, was hospitalized for circular hyperpathy and unsustainable pain at the Th7 level. Prior to admission, she received 1000 mg Solu Decortin® three times every other day, which was added to the immune system modulation drug regime with Interferon beta-1b 44 μg every third day. She experienced no relief of pain. Her initial PBS score was 11 and her EDSS score was 3.0. She was treated 5 times with 40 mg TCA every third day. The PBS score went down to 4 and the EDSS score dropped to 2 on day 16 after the last TCA administration on day 15. There was only a moderate CSF protein elevation (see Table 1); no further side effects occurred.

## Case 3

A 26-year-old female patient, diagnosed with RRMS 2 years ago, felt onset of painful dysesthesia of the trunk and all limbs 3 weeks before admission. An acute MS relapse with sensory transverse lesion of the spinal cord below the Th2 level was assumed and then treated with intravenous administration of 1000 mg Solu Decortin® without distinct improvement of her symptoms over a 3-day interval in addition to her existing daily subcutaneous copolymer I application. T1-weighted MR-images revealed a single enhancing spinal cord lesion posteriorly located in the upper thoracic spinal cord (Fig. 1). The patient developed soft signs of a Cushing syndrome due to the intravenous steroids. Intrathecal TCA was administered, since this kind of treatment does not impact endogenous peripheral cortisol secretion. Therefore, side effects of systemic high-dosage steroid application do not appear[13,14]. Before start of TCA treatment, her EDSS score was 2.5 and her PBS score was 7. After two TCA applications, the painful dysesthesia moderately declined. Then TCA application was stopped for a 4-week interval due to the long half-life of TCA in CSF. At the next appointment 4 weeks later, she still reported weak pain sensations. A single intrathecal TCA administration was performed. She felt no pain on the next day, her PBS dropped to 0, we scored with 2 on the EDSS. No side effects occurred, CSF cells decreased, and protein remained in normal range (see Table 1).

## Case 4

One year before admission, a 33-year-old female patient already complained about blurred vision, corresponding to optic neuritis, for an interval of 1 week. These visual symptoms decreased without any therapy and she did not undergo further diagnostic procedures. She was sent to the department of surgery, since she suffered from slowly increasing weakness of the right leg in combination painful itching sensations at the Th5 level. After admission, MRI of the spinal cord was performed. There were Gadolinium-enhancing lesions at the levels of C1, C4/5, and Th3 (Fig. 2). The CSF showed oligoclonal IgG bands. These clinical features correspond to RRMS[15]. Her EDSS score was 2. She felt of no reduction of pain sensations after daily intravenous 1000 mg Solu Decortin® administration during a 3-day interval. In view of the successful TCA treatment effects of the first three cases and her three Gadolinium-enhancing spinal cord lesions, intrathecal TCA application was started every third day. After four TCA applications, the PBS dropped from an initial score of 9 before the start of TCA applications to a score of 3 the day after the fourth administration; the EDSS remained unchanged. No relevant CSF changes occurred (see Table 1). We performed no further immune system modulating therapy during the treatment interval.

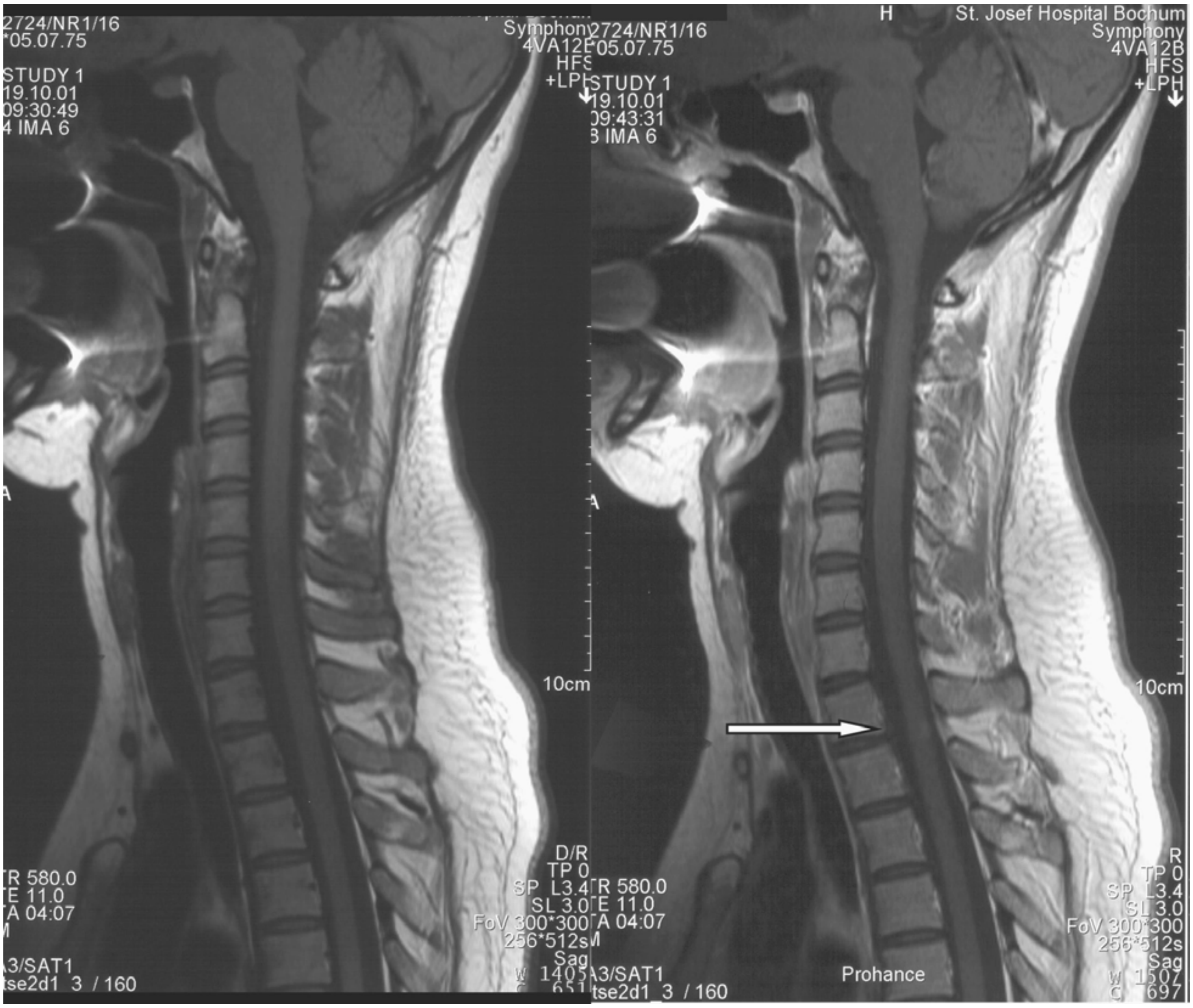

**FIGURE 1.** Sagittal T1-weighted SE images without (left) and after (right) injection of Gd-DTPA. A posteriorly located area of enhancement is visible in the upper thoracic spinal cord.

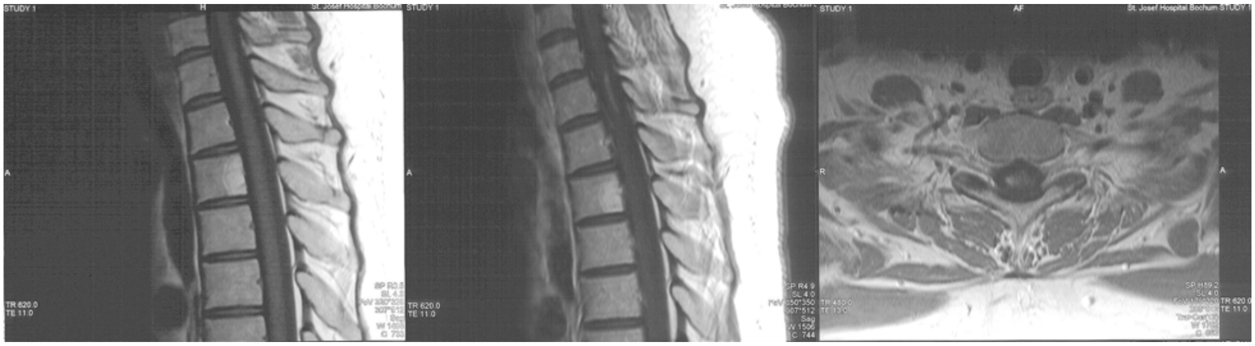

**FIGURE 2.** Sagittal pre- (left) and post- (middle) Gd-DTPA T1-weighted SE images of the thoracic spinal cord showing an enhancing area at the level of Th3. On the axial post-Gd-DTPA image (right), the enhancing lesion is clearly visible in the right lateral column of the spinal cord.

## DISCUSSION

Our results demonstrate the efficacy and safety of repeated intrathecal TCA application in only four RRMS patients. All four patients had a prior failed attempt of therapy with intravenous steroids. Each patient thus serves as their own active drug control before the experimental TCA intervention. These patients clinically experienced an acute onset of a relapse with painful sensations due to inflammatory lesions of the spinal cord, which we demonstrated in two of them by MRI imaging. Occurrence of pain is very common in MS patients and appears up to a range of 61%; 68% of the patients report that they experience conventional symptomatic pain-relieving treatment options as unsatisfactory[16]. Despite the pain-reducing efficacy of TCA administration, we stress that the first-choice treatment of an acute relapse in RRMS patients is intravenous high-dose steroid administration, which has been proven as very efficacious in various trials. However, in view of our promising case reports, we suggest that one should consider intrathecal TCA application in RRMS with acute onset of unpleasant, quality-of-life-impairing pain syndromes. Intrathecal TCA administration is advantageous in comparison with intravenous steroid application since TCA must not pass the blood brain barrier. Thus, relief of pain may hypothetically be due to the persistent higher steroid concentration at the inflammatory site of the spinal cord, which may result in onset of pain by direct or indirect affection of pain sensations transporting structures by, for example, local edema. We emphasize that intrathecal TCA application should only be performed by physicians with a broad experience of this kind of treatment after initial careful and extensive information about the patient and only with his written consent.

In conclusion, we show that repeat intrathecal TCA application in RRMS patients with acute onset of pain sensations is efficacious and safe. We suggest intrathecal TCA administration as an additional second-choice treatment option when the pain syndrome did not respond to a prior intravenous high-dosage steroid application.
